# Insurance of Children

**Published:** 1889-02-23

**Authors:** 


### The Insurance of Children.

The untiring efforts of the Society for the Prevention of Cruelty to Children, the severe but just condemnation of the system uttered by Mr. Justice Day in giving judgment on a case that was tried before him in the summer, and the recent letter of Mr. Braxton Hicks, the coroner for Surrey, to the *Times*, are all compelling public attention to the subject of infantile insurance; and will, in time, compel legislative action in a matter of the first importance to the life and well-being of the children of the poor. The insurance of young children's lives has led to an incalculable amount of crime; neglect, cruelty, and death have resulted from it. If the stern proof of these statements is not to be found in our criminal court records in the form of convictions for murder and manslaughter, the reason is that convictions are hard to get when the evidence is mostly that of circumstance and inference, and when our law, almost too rigorously just to the accused, is chary of assuming evil motives where they cannot be definitely proved.

The insurance of children's lives sprang from a desire that is almost a matter of self-respect among the poor, the wish to give any member of their family who dies a decent funeral. With them a "decent" funeral means an extravagant one; it means not merely the inevitable cost of conveyance to the grave, but "a bit of black" for the survivors, and a feast, which often becomes a drinking bout, for them and their friends. This is the reason why children's lives are insured by respectable members of the working classes, who have, however, no ideas of the dignity of thrift beyond their fellows, and cherish an almost morbid dread of seeming mean. With them the burial insurance is a form of thrift, a preparation for possible contingencies, a laying-by for a rainy day.

It does not require much reasoning power to see that it is a false form of thrift; for if the child lives the money spent in premiums is wasted, and if it dies the possession of a considerable sum of ready money is apt to develop a habit of extravagance in the management of funerals which is to be deprecated in all classes of the community. But the worst effects of this system are perceived among those classes of the poor who are not respectable. Once give these to understand that the deaths of their children will put money in their pockets, and those children are doomed. They are not killed by knife or poison, because if it was obvious that they were murdered, the insurance money would not be paid to the murderer; but the dullest criminal in the world can easily enough extinguish the feeble flame of life in a young child over whom he has complete control. Neglect, improper or insufficient food, exposure to cold or insanitary conditions, will all lay the seeds and develop the blossoms of disease in a child's constitution; and if, as is usually the case, a doctor is called in to see the child before it dies, he can only say that it succumbed to consumption, or pneumonia, or malnutrition, or whatever form of ailment death took. He may in a few cases have warned the parents or guardians of the patient that its life depended on a change in their treatment, and can testify to his warning having been neglected; but as a rule he is called in only when death is imminent, and in order that a legal certificate may be easily obtained.

The only safeguard against the abuse of child insurance is parental affection, and history has before now given proof that when the quiver is full, and the larder is empty, parental affection gives way before cupidity or hunger. But if the love of father or mother is worth anything—and it certainly is, or child insurance would come to an end; for it is from the premiums paid on the lives of those who do not die that the insurance companies make their profit—if this offers a protection, what help is there for those hapless little creatures who are left in the charge of that guardian of the orphaned or illegitimate children of the poor, the baby-farmer? The nurse, perhaps, receives a considerable sum on taking charge of the child, but one outrageously insufficient to provide it with food and clothing till it is of an age to maintain itself; it is taken on the chance that it will die soon enough to leave a residue of profit to the guardian from the sum entrusted to her; and in such cases the chance becomes a certainty, made all the more assured if the child's life is insured. Or weekly payments may be given. In this case the child has a better chance; for some report of its health must be given, and an occasional visit from its mother may be anticipated. But too often the mothers are anxious only to forget that the child is theirs; they fall in arrears with their payments for some reason or another, and then the object of the baby-farmer is to secure the death of her charge soon enough for the insurance money—£6 to £10—to recoup any expense she may have incurred for it.

In most offices, before an insurance is granted to one person on the life of another, the person seeking the policy is required to prove that he has an "insurable interest" in that other's life—that is, that he is more likely to profit by the life than the death of the person he wishes insured. A rich man would find a difficulty in insuring the life of his child; a guardian would find it all but impossible to effect an insurance on the life of his ward. Indeed, an Act of George III. prohibits all insurances on lives where the person who asks for the policy has not this insurable interest; but, as if to facilitate crime, Parliament repealed this prohibition in the case of persons insured under the Friendly Societies' Act of 1875—an Act which chiefly concerns the poor. We do not imply that family affection is rarer among the poor than among the rich; most of us know of cases where the parents stint themselves in order that the children may not want. But if a rich man does not love his child he is not tempted to let it die to save the cost of its food, and secure a ten-pound note.

And, as was said before, death is so easily managed, and so difficult to prove as done intentionally. Insufficient feeding may be due to poverty, improper feeding to ignorance. How can either judge or jury, doctor or coroner, say whether or not a mother knew that in giving her infant a "bit of what's going," from pickled pork to raw whisky, she was doing her best to kill it? Ignorance is so common that it is difficult to say where it ends and malice begins; and the unscrupulous benefit by the existing doubt. They have learnt that if you choose food unsuitable for the nutrition of a young child, the more you give it the less it is profited; it is easy to kill by apparent kindness, and the law cannot touch you for it. Occasionally the murder is managed in a bungling manner, or accompanied by such open cruelty as to arouse suspicion, and the murderer may be punished; but such cases are so rare as to exercise but little deterrent effect on others who incline to similar crimes. The only way in which these can be prevented is by the Legislature altering the laws relating to infantile insurance in a such a fashion that no one who would presumably profit by the death of a child should be permitted to effect an insurance on its life.

Soon after Justice Day expressed his condemnation of the present system, an insurance journal published an irate article on the subject. It said that this question was one to be decided, not by doctors and judges, but by insurance companies. There was no argument stated, beyond the somewhat inconclusive one that "It was all nonsense;" but the inferential one was that if children whose lives were insured died with such appalling frequency, it would not pay the companies to effect insurances on them. That the present system does pay is evident from the fact that the companies can afford to give from 48 to 54 per cent. in commission to the agents who secure them subjects. This only proves that—not counting the policies that lapse because the premiums cease to be paid; a fertile source of income in all such associations—there are parents who do their duty to their children without thinking of possible profit from their death; it does not prove that those who are evilminded cannot find a fiendish gain in the deaths of children they neglect and hate. What is necessary is a reform that will make these life insurances truly insurances of life, not of death.

In France there is such a system practised. Parents invest a fixed sum at the birth of a child, and make yearly payments during its childhood in order that when it grows up it may have a little money to begin the world with—something that will pay for the son's apprenticeship, or furnish the daughter's *dot*. If the child dies before the age fixed for payment of the money all premiums are forfeited, a consideration that would probably prevent English parents, already selfish and improvident, from taking up such a scheme. Mr. Braxton Hicks suggests a plan that has a double merit, in providing for both the life and the death of the child insured.

By way of prevention of the evils of the present system he advises that only parents should be allowed to insure, and that the companies should appoint an undertaker to conduct the funerals of all children whose lives have been insured with them; thus the parents would receive no cash payments, and one great temptation to crime would be removed. On the other hand, he suggests "that if the child attains the age of sixteen years it should be compulsory on insurance companies to give a surrender value for the policy." This would encourage thrift in the parent, for by the time the child was 16 years old it would, presumably, be able to contribute towards its own keep, and the parent could then recoup himself for the expenses he might have been put to in insuring against the possible death of his child, or, should he be so minded, he might use the money for furthering the child's interest.

These are simple and effectual remedies for the present state of things, remedies that appeal to the common sense of the public. And while it is the province of the Legislature to make and alter laws, it is the common sense of the public that must impress on it the necessity for action. To this public—the public of parents and lovers of children—we make our appeal.

